# Social and occupational functioning scale for epilepsy: performance in Brazilian adult patients with epilepsy

**DOI:** 10.1055/s-0045-1811721

**Published:** 2025-10-31

**Authors:** Gloria M. A. S. Tedrus, Danilo Wingeter Ramalho, Elisa Dal Rio Teixeira

**Affiliations:** 1 Pontifícia Universidade Católica de Campinas, Campinas SP, Brazil.

**Keywords:** Employment, Epilepsy, Seizures, Social Interaction

## Abstract

**Background:**

Epilepsy is a condition that can lead to social difficulties and restrictions.

**Objective:**

To evaluate social and occupational functioning and relationships with clinical findings of Brazilian adult patients with epilepsy.

**Methods:**

The scores of the Social and Occupational Functioning Scale for Epilepsy (SOFSE) were related to the clinical data of adult patients with epilepsy.

**Results:**

Among the 79 cases included, with a mean age of 44.3 years old, 47 were employed and 32 were unemployed. It was observed that there was a predominance of males among the employed patients (32 [68.1%] versus 15 [31.9]; *p* = 0.008), most patients had a companion (27 [57.4%] versus 20 [42.6%]; *p* = 0.005), and most patients had no anxiety symptoms on the Hospital Anxiety and Depression Scale (HADS) (30 [63.8%] versus 17 [36.2%]; *p* = 0.011). The total score in the SOFSE was 64.7, with a higher total score among the employed patients (56.6 ± 12.5 versus 70.3 ± 14.3; *p* < 0.001). There was a correlation between the total SOFSE score with formal education (r = 0.30) and with HADS-anxiety (r = - 0.56) and HADS-depression (r = - 0.36). Lower scores in the communication domain were associated with a high frequency of seizures. There were lower scores in the domain leisure activity in TLE (2.0 ± 2.0 versus. 2.2 ± 1.5; *p* = 0.014).

**Conclusion:**

The unemployment rate was high in adults with epilepsy. Employment was associated with male gender, having a companion, and absence of anxiety symptoms. Better functional adjustment was associated with schooling. Social and occupational functional impairment was associated with a high frequency of seizures, TLE, and anxious and depressive symptoms.

## INTRODUCTION

In epilepsy, there may be difficulties in family and social interaction and lower marriage rates with impairment of the integrated perspective of biological and social health. 1 2 3 4 5 6 The impairment in social relationships in epilepsy is greater when compared with the impairment observed in other chronic diseases; however, there is a great difference between populations and cultural conditions. 7 The perception of stigma and the fear of being rejected or devalued are some of the factors that contribute to social isolation in epilepsy. 8

It is well known that having a job or being employed is a relevant aspect of social integration and the perception of individuals' inclusion. Various social conditions and clinical aspects, such as depression and anxiety, among other psychiatric disorders, can result in substantial functional impairment and cause economic impact, such as absenteeism, reduced productivity at work, and unemployment. However, unemployment and informal employment rates with low-skilled activity are higher in epilepsy than in the general population. 5 9 Epilepsy variables, such as the unpredictability of seizures, have a negative impact on social and emotional life and can lead to limitations in work insertion and professional integration.

A systematized evaluation with a validated scale of occupational aspects in epilepsy can contribute to better knowledge and bring new data in the social area of epilepsy. The present study aimed to evaluate social and occupational functioning in adult patients with epilepsy and to relate it to demographic data and clinical variables.

## METHODS

The present study was conducted at the clinical neurology outpatient clinic of the PUC-Campinas Hospital in Campinas, state of São Paulo, Brazil, from September 2022 to December 2023. This hospital assists patients referred by the Basic Health Units of the Unified Health System and serves, in most cases, individuals of low socioeconomic and cultural levels.

Patients aged between 18 and 60 years old with a diagnosis of epilepsy for at least 2 years and in regular use of antiseizure medication (ASM) were consecutively included. The diagnosis of epilepsy was based on the criteria of the International Classification of Epilepsies and Epileptic Syndromes of the International League Against Epilepsy. 10 Patients with progressive neurological diseases, moderate/severe cognitive disorders, neoplastic and disabling diseases, and other health conditions that compromised functionality were excluded. The research project was approved by the Human Research Ethics Committee of PUC-Campinas (CAAE: 13195619300005481; No.: 5.507.182. July 04, 2022).

### Procedures

The patients were evaluated on the regular day of the medical appointment, and all instruments were applied in sequence and individually in an appropriate hospital room. Demographic aspects (age, sex, education, and marital status) and clinical data of epilepsy (age at onset, type and frequency of seizures, and number of ASMs in use) were evaluated. Electroencephalogram (EEG) and imaging data from the hospital's medical records were used. The condition of employment/occupation was assessed in the previous 3 months and was classified as employed, unemployed, or domestic work (only activities in the home and unpaid). Unemployment was defined as the absence of work with regular income.

The following instruments were applied:

- Hospital anxiety and depression scale (HADS) 11 12 to assess the presence of depressive (HADS-depression) and anxiety (HADS-anxiety) symptoms, and the cutoff score was used according to the criteria validated in Brazil;
- Social and Occupational Functioning Scale for Epilepsy (SOFSE). 13

The scale is composed of 30 items related to the main functional difficulties in the daily life of individuals.

The scale is composed of six domains, comprising paid work and unpaid work:

- interpersonal relationships;
- communication;
- social activities;
- leisure activities;
- instrumental living skills, and
- occupation.

The score ranges from 0 to 100; the higher the value, the better the individual's functional status. The scale is specific to epilepsy and has been translated and adapted into Brazilian culture. 14

### Statistical analysis

The present study evaluated the demographic data of occupational status (employed, unemployed), possession of a driver's license (yes/no and type of license), and the SOFSE scores (domains and total score) of adult patients with epilepsy. For the evaluation of the HADS, the cutoff point was used according to the criteria validated in Brazil (HADS-anxiety ≥ 8 in the HADS-depression > 7).

Exploratory data analysis used summary measures (mean, standard deviation [SD], first quartile, median, third quartile). The SOFSE scores were related to the demographic aspects (age, education, sex, and marital status) and clinical aspects (age at the time of the first seizure, duration of epilepsy, frequency and type of seizures, type of epilepsy and syndrome, and the number of ASMs in use) and the HADS scores (HADS-anxiety and HADS-depression). The Spearman correlation test was applied between the SOFSE scores (domains and total score) and the continuous variables. The association between SOFSE scores and categorical variables was performed using the Wilcoxon or the Mann-Whitney test. Factors associated with employment/unemployment were assessed using simple and multiple logistic regression. The stepwise criterion was used to select factors in the multiple analysis. A significance level of 5% was adopted. The Statistical Packages for Social Sciences, version 27.0 (SPSS, IBM Corp.) software was used.

## RESULTS

In the present study, a consecutive review of the current working conditions and social and occupational functioning of 79 patients, 56% female, aged between 20 and 82 years old and with a mean age of 44.3 ± 13 years old. The age at the time of the first seizure was 21.3 ± 15.4 years old. The total score in the SOFSE was 64.7 ± 15.1. A total of 25% of cases had a driver's license, with lower values in female patients.

The demographic and clinical variables of the 79 patients according to their occupational status are shown in Tables 1 and 2 . There was a difference in occupational status according to sex, marital status, and the presence of anxiety symptoms. It was observed that male patients were more employed, had more companions, and had a higher rate of possession of a license to drive vehicles. No significant difference was observed in epilepsy variables according to occupational status (employed versus unemployed).

**Table 1:** Demographic and clinical data and HADS scores according to employment status in 79 patients with epilepsy

		Unemployed ( *n* = 32)	Employed ( *n* = 47)	*p-value*
Gender, *n* (%)	Male ( *n* = 44)	12 (37.5%)	32 (68.1%)	0.008*
	Female ( *n* =35)	20 (62.5%)	15 (31.9%)	
Marital Status	Single, divorced, or widower (n = 44)	24 (75.0%)	20 (42.6%)	0.005*
	With companion (n = 35)	8 (25.0%)	27 (57.4%)	
Seizure frequency	> 1x/ month (n = 13)	7 (21.9%)	6 (12.8%)	
	1x/month (n = 22)	12 (37.5%)	10 (21.3%)	0.968
	1-11x/year (n = 22)	8 (25.0%)	14 (29.8%)	0.316
	Free 1 year or more (n = 22)	5 (15.6%)	17 (36.2%)	0.068
Type of seizure	Focal (n = 51)	21 (65.6%)	30 (63.8%)	0.870
	Generalized (n = 28)	11 (34.4%)	17 (36.2%)	
Epileptic syndrome	Structural (n = 58)	25 (78.1%)	33 (70.2%)	
	Genetic (generalized) (n = 8)	2 (6.25%)	6 (12.8%)	0.339
	Unknown (n = 13)	5 (15.6%)	8 (17.0%)	0.760
Type of epilepsy	Other epilepsies (n = 60)	28 (87.5%)	32 (68.1%)	0.055
	TLE-HS (n = 19)	4 (12.5%)	15 (31.9%)	
No. of ASM in use	One (n = 46)	18 (56.2%)	28 (59.6%)	0.769
	≥ 2 (n = 33)	14 (43.8%)	19 (40.4%)	
HADS-anxiety (total score)	No (< 8) (n = 41)	11 (34.4%)	30 (63.8%)	0.011 *
	Yes (≥ 8) (n = 38)	21 (65.6%)	17 (36.2%)	
HADS-depression	No (≤ 7) (n = 43)	17 (53.1%)	26 (55.3%)	0.848
	Yes (> 7) (n = 36)	15 (46.9%)	21 (44.7%)	

Abbreviations: ASM, antiseizure medications; HADS, Hospital Anxiety and Depression Scale; TLE-HS, temporal lobe epilepsy with hippocampal sclerosis.

Note: * *p < 0.05.*

**Table 2:** Demographic and clinical data and scores in the HADS and SOFSE according to the employment status of 79 patients with epilepsy

Variable	Unemployed ( *n* = 32)		Employee ( *n* = 47)		*p-value*	OR	LI	LS
	Average (p.p.)	Median [Q1; Q3]	Average (p.p.)	Median [Q1; Q3]				
Age (years old)	46.1 (15.0)	47.5 [34.0; 57.2]	43.1 (11.6)	42.0 [32.0; 51.5]	0.313	0.98	0.95	1.02
Formal education (years)	7.59 (2.99)	8.00 [4.00; 11.0]	8.43 (3.49)	8.00 [5.50; 11.0]	0.271	1.08	0.94	1.25
Age at the time of the first seizure (years old)	21.0 (18.0)	15.5 [12.2; 26.8]	21.5 (13.7)	17.0 [12.0; 28.0]	0.880	1.00	0.97	1.03
Duration of epilepsy (years)	25.1 (16.3)	23.0 [10.0; 38.2]	21.6 (13.1)	22.0 [10.5; 28.5]	0.286	0.98	0.95	1.01
HADS-anxiety	9.28 (4.24)	9.50 [6.00; 12.0]	7.04 (4.44)	7.00 [4.00; 11.0]	0.032*	0.89	0.79	0.99
HADS-depression	6.44 (3.75)	5.50 [3.00; 9.00]	6.11 (3.73)	6.00 [3.00; 9.00]	0.696	0.98	0.86	1.10
SOFSE - total score	56.6 (12.5)	56.0 [51.8; 65.2]	70.3 (14.3)	67.0 [61.5; 82.5]	< 0.001*	1.08	1.04	1.14
Interpersonal relationships	9.25 (2.44)	10.0 [7.75; 11.0]	9.74 (2.47)	11.0 [8.50; 12.0]	0.378	1.09	0.90	1.31
Communication	9.28 (3.18)	9.50 [7.00; 12.0]	11.3 (3.18)	12.0 [10.0; 14.0]	0.010*	1.22	1.06	1.43
Social activities	8.47 (2.49)	9.00 [7.00; 10.0]	8.62 (3.08)	9.00 [6.00; 11.0]	0.819	1.02	0.87	1.20
Leisure activities	1.94 (1.83)	2.00 [0.00; 3.00]	2.06 (1.95)	2.00 [0.00; 3.00]	0.769	1.04	0.82	1.33
Instrumental living skill	19.3 (6.56)	19.5 [15.0; 24.2]	20.4 (6.81)	20.0 [15.0; 26.0]	0.479	1.02	0.96	1.10
Occupation – paid work	0.00 (0.00)	0.00 [0.00; 0.00]	11.4 (5.02)	13.0 [12.0; 14.5]	NC			
Occupation – unpaid work	8.38 (3.56)	9.00 [6.00; 11.0]	6.70 (5.14)	7.00 [0.00; 10.5]	0.116	0.92	0.83	1.02

Abbreviations: HADS, Hospital Anxiety and Depression Scale; SOFSE, Social and Occupational Functioning Scale for Epilepsy.

Note: * *p < 0.05.*

### SOFSE: demographic and clinical data, HADS, occupational situation

In the SOFSE, the total score and the communication dimension were significantly higher in the employed patients. The other dimensions had no significant difference according to the occupational situation ( Table 2 ).

**Figure 1:**
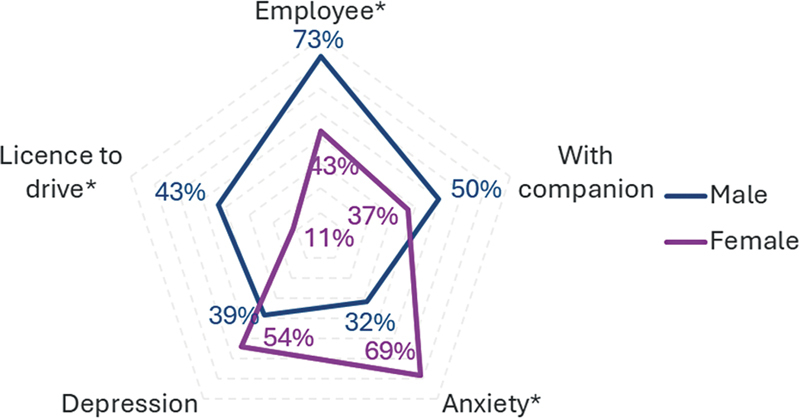
Note: *Variables with statistical differences between the sexes. Clinical and demographic aspects between the sexes.

There was a difference in the SOFSE scores according to sex. It was observed that the percentage of variation was negative in female patients compared to that observed in males ( Figure 1 ). There were differences between the sexes in the dimensions: occupation - paid work (- 43%) and occupation - unpaid work (- 31%), communication (- 18%), leisure activities (- 18%), total score (- 15%), social activities (- 7%), and interpersonal relationships (- 4%) (Wilcoxon test).

In the analysis of the comparison between the employed and unemployed groups about the continuous and categorical variables evaluated individually, it was observed that a one-point increase in HADS-anxiety reduces the chance of patients being employed by 12%. A one-point increase in the total SOFSE score increases the chance of patients being employed by 8%. A one-point increase in the communication domain increases the chance of patients being employed by 22%. Male patients are 3.56 times more likely to be employed than female patients. Patients with a partner are 4.05 times more likely to be employed than those without one. Patients without anxiety are 3.37 times more likely to be employed than people with anxiety.

The demographic and clinical variables and the SOFSE scores that presented a *p* -value ≤ 0.1 in the individual analysis were placed in a multiple logistic regression model with stepwise variable selection criteria. This model showed that the variables that together best predict occupation are the SOFSE total score ( *p* < 0.001; odds ratio [OR] = 1.09 [1.04–1.14]) and the frequency of seizures ( *p* = 0.049; OR = 3.51 [1.07–13.44]). Patients with higher SOFSE scores and controlled seizures are more likely to be employed.

### SOFSE scores according to clinical and demographic variables

Age was negatively correlated with the instrumental living skill dimension. There was a correlation between age at the first seizure and the dimensions of interpersonal relationships and leisure activities. Higher formal education was correlated with the SOFSE (total score) and the instrumental living skill dimension.

There was a significant negative correlation between HADS-anxiety and SOFSE (total score) and all dimensions. There was a significant negative correlation between HADS-depression and SOFSE (total score) and the dimensions of interpersonal relationships, communication, instrumental living skills, occupation–paid work, and occupation–unpaid work. There was no significant correlation between other numerical variables ( Table 3 ).

**Table 3:** Correlation between SOFSE and clinical and demographic variables

	SOFSE domains							
	SOFSE (total score)	*Interpersonal relationships*	*Communication*	*Social activities*	*Leisure activities*	*Instrumental living skill*	*Occupation – paid work*	*Occupation – unpaid work*
Age (years old)	- 0.21	0.03	0.09	0.06	- 0.06	- 0.31*	- 0.11	- 0.16
Education (years)	0.30*	0.03	0.13	0.05	0.00	0.28*	0.18	0.11
Age at the time of the first seizure (years old)	- 0.09	0.14*	0.01	- 0.04	-0.01*	- 0.04	- 0.18	- 0.05
Duration of illness (years)	- 0.04	- 0.16	0.04	0.07	0.00	- 0.19	0.15	- 0.06
HADS-anxiety	- 0.56*	- 0.39*	- 0.37*	- 0.32*	- 0.08	- 0.29*	- 0.24*	- 0.38*
HADS- depression	- 0.36*	- 0.35*	- 0.27*	- 0.12	0.07	- 0.32*	- 0.16	- 0.24*

Abbreviations: HADS, Hospital Anxiety and Depression Scale; SOFSE, Social and Occupational Functioning Scale for Epilepsy.

Notes: Spearman's correlation * *p < 0.05.*

Patients with scores ≥ 8 on the HADS-anxiety have lower scores in the domain of interpersonal relationships. Scores in the communication domain were associated with seizure frequency and with scores in HADS-anxiety. Patients with THE-HS had lower scores in the leisure activities dimension. Patients with scores > 7 in the HADS-depression had lower scores in the instrumental living skill domain. Patients with a companion had higher scores in the occupation–paid work domain. There was no significant difference in the SOFSE scores (total score and dimension) according to other categorical variables ( Table 4 ).

**Table 4:** Data on the domains of the SOFSE according to clinical variables

SOFSE- domains	Epilepsy variables		Average (p.p.)	Q1	Median	Q3	*p-value*
Interpersonal relationships	HADS-anxiety	No (< 8)	10.4 (2.2)	10.0	11.0	12.0	0.001*
		Yes (≥ 8)	8.6 (2.4)	7.3	9.0	10.0	
Communication	Seizure frequency	> 1x/month	8.6 (3.7)	5.0	10.0	11.0	0.047*
		1x/month	9.7 (3.0)	8.0	10.0	11.0	
		1-11x/year	11.9 (2.5)	10.0	12.0	13.8	
		Free ≥ 1 year	11.0 (3.5)	8.0	12.0	14.0	
	HADS-anxiety	No	11.8 (2.9)	10.0	12.0	14.0	0.012*
		Yes	9.1 (3.2)	7.0	10.0	11.0	
Leisure activities	Epilepsies	THE-HS	2.0 (2.0)	0.0	2.0	3.0	0.014*
		Other	2.2 (1.5)	1.0	2.0	3.0	
Instrumental living skill	HADS- depression	No (≤ 7)	22.0 (6.2)	16.0	22.0	27.5	0.049*
		Yes (>7)	17.5 (6.5)	14.0	16.0	21.0	
Occupation – paid work	Marital status	Unaccompanied	4.4 (6.5)	0.0	0.0	12.2	0.011*
		With companion	9.9 (6.0)	4.5	13.0	14.0	

Abbreviations: HADS, Hospital Anxiety and Depression Scale; SOFSE, Social and Occupational Functioning Scale for Epilepsy; TLE-HS, temporal lobe epilepsy with hippocampal sclerosis.

Note: * *p < 0.05.*

## DISCUSSION

The consecutive sample evaluated was composed of 79 adult patients with epilepsy in outpatient care, aged within the usual working age range, with low formal education, and it was observed that 40% of the cases did not have paid occupational activity. Low formal education and impairment of professional qualification and employment status are sociodemographic factors frequently described in epilepsy, with consequences on social and family competence and considerable negative economic impact, which is sometimes devastating. 1 4 14 15 16 17 18 A recent review study described that the average employment rate in epilepsy is 58%, with values adjusted according to the standard definition of the Bureau of Labor Statistics of the International Labor Organization and without significant differences between continents. 15 Data on the relationship between epilepsy and employment in recent studies in Europe and from a Brazilian group confirm that changes in legislation and the creation of specific programs in the health area can contribute to the social and occupational integration of adult patients with epilepsy 19 20

When assessing marital status, it was observed that most patients did not have a companion. Similar findings have been reported in clinical studies on epilepsy across different cultures, and they may be related to stigma and the significant difficulties in establishing social and affective relationships observed in this condition. 16 19

There was a significant relationship between occupational status and the demographic and clinical variables evaluated, and it was observed that the largest number of individuals with paid occupational activity were male, had a companion, and had a lower occurrence of anxiety. These data reinforce the differences between the sexes and the double stigma perceived by women with epilepsy. The relationship between occupational status and the occurrence of anxiety symptoms may be a two-way relationship.

The licensing rate to drive vehicles was lower in both sexes than the data in the Brazilian population and significantly lower in women. These data confirm the mobility and independence difficulties described in epilepsy. Similar values were expressed in another recent study in which 305 patients with epilepsy were evaluated. 22

No statistically significant association was identified between the type and frequency of epileptic seizures and occupational status, in line with the findings of a systematic review that included 95 studies and demonstrated that the adjusted employment rate is similar between individuals with controlled and uncontrolled seizures. 15 . However, other clinical factors, such as physical limitations, behavioral changes, and cognitive dysfunction, may have a more substantial impact on work capacity, professional trajectory, and financial earnings than seizure type or frequency. 17

### SOFSE scale: Demographic and clinical data

In this sample, the SOFSE values suggest low or regular performance of capacity and functionality in the activities of daily living of patients with epilepsy. Similar to the findings described in other studies, there was low involvement and adherence to leisure activities, which suggests that patients with epilepsy have low social interaction, little participation in leisure-time physical activities, and limitations in various aspects of social life, possibly related to the fear of having seizures and the feeling of stigma. 13 14

The degree of functionality and the data on the instrumental living skill, leisure activities, and occupation (paid work and unpaid work) dimensions differed according to sex, age, marital status, and formal education, which suggests that the perception of better functional, social, and occupational status in daily activities is influenced by demographic aspects in epilepsy.

It was observed that functional impairment in the SOFSE (total score and dimensions) was associated with earlier age at the time of the first seizure with epilepsy variables such as seizure frequency (with and without seizures in the last year), type of epilepsy (temporal lobe epilepsy with hippocampal sclerosis versus other epilepsies) and the presence of psychiatric comorbidities (depressive or anxious symptoms). These data confirm the significant multidimensional impact and the functional and occupational impairment of epilepsy variables when using an appropriate quantitative/qualitative instrument.

There are limitations to the present study. The study was conducted in a single center with a small sample, which limits the accuracy of the results and makes it difficult to generalize the data. This is a cross-sectional study with limitations related to the type of study. In the present study, there is an inherent bias when using self-report questionnaires. However, on the other hand, the data obtained are new when using the SOFSE, a Chinese scale still not used in international studies.

In conclusion, the use of a specific scale for functional and occupational assessment confirmed the presence of impairment of social and occupational functioning in epilepsy and its relationship with demographic, clinical, and psychosocial factors, which reinforces the need for effective measures of professional and occupational integration in epilepsy.
